# Absorption of the [bmim][Cl] Ionic Liquid in DMPC Lipid Bilayers across Their Gel, Ripple, and Fluid Phases

**DOI:** 10.1021/acs.jpcb.2c00710

**Published:** 2022-04-26

**Authors:** Antonio Benedetto, Elizabeth G. Kelley

**Affiliations:** † Department of Science, University of Roma Tre, 00146 Rome, Italy; ‡ School of Physics, and Conway Institute of Biomolecular and Biomedical Research, University College Dublin, Dublin 4, Ireland; § Laboratory for Neutron Scattering, Paul Scherrer Institute, 5232 Villigen, Switzerland; ∥ NIST Center for Neutron Research, National Institute of Standards and Technology, Gaithersburg, Maryland 20899, United States

## Abstract

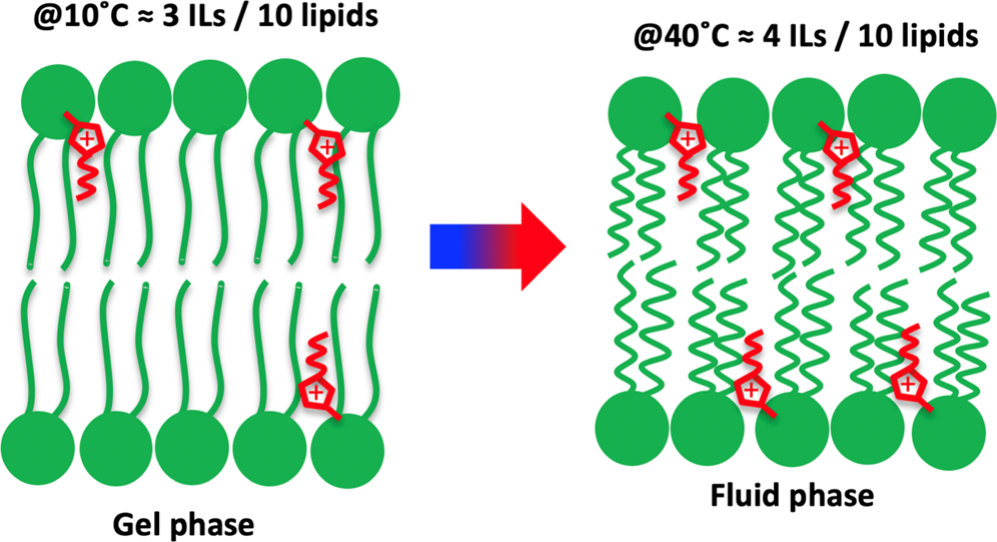

Lipid bilayers are a key component of cell membranes and play a crucial role in life and in bio-nanotechnology. As a result, controlling their physicochemical properties holds the promise of effective therapeutic strategies. Ionic liquids (ILs)—a vast class of complex organic electrolytes—have shown a high degree of affinity with lipid bilayers and can be exploited in this context. However, the chemical physics of IL absorption and partitioning into lipid bilayers is yet to be fully understood. This work focuses on the absorption of the model IL [bmim][Cl] into 1,2-dimyristoyl-*sn*-glycero-3-phosphocholine (DMPC) lipid bilayers across their gel, ripple, and fluid phases. Here, by small-angle neutron scattering, we show that (i) the IL cations are absorbed in the lipid bilayer in all its thermodynamic phases and (ii) the amount of IL inserted into the lipid phase increased with increasing temperature, changing from three to four IL cations per 10 lipids with increasing temperature from 10 °C in the gel phase to 40 °C in the liquid phase, respectively. An explicative hypothesis, based on the entropy gain coming from the IL hydration water, is presented to explain the observed temperature trend. The ability to control IL absorption with temperature can be used as a handle to tune the effect of ILs on biomembranes and can be exploited in bio-nanotechnological applications.

## Introduction

Cell membranes play a key role in life. They are composed of a variety of proteins, protein complexes, and several other molecules, such as saccharides, all of which are embedded in a bilayer structure composed of hundreds of different lipids.^1−3^ The chemical and physical properties of this lipid bilayer regulate a variety of processes, including membrane protein function, cell recognition by the immune system, and cell division.^4−7^ For example, the inhomogeneous distribution of lipids along the bilayer surface is at the origin of the well-known raft domains, in which proteins and other molecules cluster to carry out specific biochemical functions.^8,9^ On the other hand, the asymmetric distribution of lipids between the two bilayer leaflets is key to several signaling pathways, including cell apoptosis.^10,11^ Moreover, tumor cells have different lipidomic compositions in comparison to their healthy counterparts, and lipid composition controls the fluidity and viscoelastic properties of the membrane, which in turn alters protein functions, cell division, and cell migration.^12−15^ As a result, alterations of the cellular lipid membrane composition and organization can lead to cell malfunction, as observed in several pathological conditions, including cancer.^16−18^ In this context, several novel therapeutic strategies targeting the lipid components of cell membranes have been proposed.^19^ From a different perspective, lipid-based nanoparticles are also used as carriers for drug delivery.^20−27^ A very timely example is provided by the new mRNA-based Covid-19 vaccines, in which the mRNA segments—containing the information to build the Spike proteins—are encapsulated in lipid nanoparticles (LNPs).^28−33^ It is then the affinity of these lipid nanoparticles with the cellular lipid membranes in our bodies that enables the diffusion of the mRNA segments into the cytoplasm. In summary, lipid bilayers are involved in several key processes at the cellular level and used in a variety of bio-nanotechnological applications. As a result, being able to control the physicochemical properties of these supramolecular structures holds the promise of effective therapeutic approaches. In this context, ionic liquids (ILs) can play a novel and important role.^34−36^

ILs are a relatively new and vast class of organic electrolytes composed of an organic cation and either an organic or inorganic anion. They possess a variety of intriguing properties, including being liquid around room temperature and having a low vapor pressure, which can be controlled by tuning their chemistry.^37,38^ Motivated by the potential use of ILs at the industrial scale, several studies have focused on their cytotoxicity, which was found to range from moderate to high depending on the IL.^39^ Cytotoxicity, however, requires affinity. This has motivated, in turn, a series of investigations aimed at understanding the chemical–physical origins of these interactions, to be then exploited in applications.^35,40−45^ In this context, several biochemical and chemical physics studies on the effects of ILs on proteins, biomembranes, saccharides, and cells have been carried out in the last decade.^46,47^ It has been shown, for example, that ILs are able to stabilize proteins,^48,49^ to either favor or inhibit the formation of amyloids,^50,51^ to disrupt biomembranes,^52−55^ and to kill bacteria and cancer cells at doses that are not lethal to healthy cells.^56,57^ Among all of these studies, one of the major focuses has been devoted to the investigation of the interactions between ILs and model biomembranes, mimicked by lipid bilayers.^52−54,58−66^ It has been shown, in this context, that IL cations, dispersed at low doses at the water–bilayer interface, diffuse into the lipid region of the bilayer,^61,62^ causing variations in the lipid dynamics^63,64^ and bilayer mechanoelasticity.^45,62,65,66^

Reasonably, these observed effects on lipid bilayers are directly connected to the amount of IL absorbed in the lipid bilayer. However, very little is known about IL absorption in these systems. Although the IL concentration in a solution can be easily controlled, there are no studies that link the IL concentration in solution to the IL concentration absorbed in the lipid phase. This latter, however, is the key information. Knowing the IL concentration in the lipid phase as a function of its concentration in solution, system temperature, lipid phase, and lipid and IL types is necessary to facilitate basic and applied research in the field. To start to tackle this lack of knowledge, we present here the first set of experimental data showing the effect of temperature and lipid thermotropic phase on IL absorption in lipid bilayers at a fixed IL concentration in the solvent phase. To do so, small-angle neutron scattering (SANS) was employed to investigate the absorption of one of the most studied ILs, i.e., [bmim][Cl] (1-butyl-3-methylimidazolium chloride), in 1,2-dimyristoyl-*sn*-glycero-3-phosphocholine (DMPC) lipid vesicles dispersed in water, at a concentration below the critical micellar concentration (CMC) of the IL. DMPC has been chosen among other lipids and lipid mixtures because (i) PC-based lipids are the most abundant lipid type in cell membranes, and (ii) among the other PC-based lipids, DMPC is fluid at physiological temperatures.

SANS is a widely used neutron scattering technique to determine nanoscale structures.^67−71^ Its basic principles are very similar to those of small-angle X-ray scattering (SAXS); however, their ability to resolve structures differs, making them two complementary techniques.^72−76^ SANS has been successfully used to study lipid vesicles in different environments and conditions as, for example, to determine (i) the effect of temperature, pressure, and pH on the phase behavior of (DMPC) lipid vesicles;^3,77−82^ (ii) the degree of asymmetry and domain formation in mixed lipid vesicles;^83−89^ (iii) the absorption of antimicrobial peptides and drugs in lipid vesicles;^90−95^ and (iv) the structure of lipid-based drug-delivery carriers. Several recent reviews provide a good overview of the use of SANS in biophysics.^96−101^ However, even though SANS has been used in many studies of lipid vesicles, there are none—to the best of our knowledge—on the absorption of ILs, making our present study the first SANS investigation of this kind.

The neutron scattering length—the ability to “see” a chemical element—depends on the composition of the atomic nuclei, making neutrons sensitive to different isotopes of a given element. This ability is unique for neutrons and has been exploited in a variety of investigations.^102−105^ We have taken advantage of it in the present study. For instance, to determine the IL absorption with the best spatial resolution achievable, the experiment was designed to have the highest possible neutron scattering contrast between the IL and the lipid tails. This was achieved using tail-deuterated lipid (d_54_-DMPC) and heavy water (D_2_O) as the solvent. The tail-deuterated d_54_-DMPC vesicles have significant neutron scattering contrast between (i) headgroups and acyl chains, (ii) headgroups and D_2_O, and (iii) acyl chains and IL, providing the best spatial resolution condition to resolve the structure of the lipid vesicle itself (i.e., outer heads–2 tails–inner heads) and measure the IL absorption.

## Materials and Methods

1,2-Dimyristoyl-*sn*-glycero-3-phosphocholine (DMPC) lipid powders were purchased from Avanti Polar Lipids (Alabaster, AL) and used without further purification. Two different lipid variants were used: (i) a fully protiated lipid (h-DMPC) and (ii) a tail-deuterated lipid (d_54_-DMPC), with main gel-to-fluid phase transition temperatures of *T*_M_ = 24°C and *T*_M_ = 20°C, respectively. DMPC can also form a ripple phase, with a gel-to-ripple phase transition temperature of *T*_M′_ = 14 °C.^106,107^ The 1-butyl-3-methylimidazolium chloride ([bmim][Cl]) ionic liquid was purchased from IoLiTec, Germany, and used without further purification. The CMC of this IL in water is 5 mol/L (hereafter indicated by M).^52^ D_2_O 99.9% was purchased from Cambridge Isotope Laboratories (Andover, MA). Table 1 reports the list of the materials used and some of their properties relevant in this study, e.g., volume and scattering length density (SLD).

**Table 1:** Theoretical Scattering Length Densities (SLD) Calculated by Dividing the Neutron Scattering Length, *b*, of Each Molecular Component by Its Volume at 30 °C^a^

material	formula	volume (Å^3^)	*b* (10^–4^ Å)	SLD (10^–6^ Å^–2^)
h-DMPC	C_36_H_72_NO_8_P	1101	3.1	0.282
h-DMPC, tails	C_26_H_54_	754	–2.91	–0.386
d54-DMPC	C_36_D_54_H_18_NO_8_P	1101	59.31	5.387
d54-DMPC, tails	C_26_D_54_	754	53.3	7.069
DMPC, head	C_10_H_18_NO_8_P	347	6.01	1.732
d54-DMPC:h-DMPC	n/a	n/a	n/a	4.85
d54-DMPC:h-DMPC, head	n/a	n/a	n/a	1.732
d54-DMPC:h-DMPC, tails	n/a	n/a	n/a	6.287
heavy water	D_2_O	30	1.9	6.35
[Bmim][Cl]	C_8_H_15_N_2_Cl	268.57	2.538	0.951
[Bmim]^+^	C_8_H_15_N_2_	167	1.580	0.94

a The neutron scattering length, *b*, of each molecular component has been computed by summing up together the coherent neutron scattering cross-sections of each chemical element in the component. The d_54_-DMPC:h-DMPC lipid mix SLDs are for the 9:1 lipid ratio used in this study.

The h- and d_54_-DMPC powders were mixed at the desired ratio and co-dissolved in chloroform at a concentration of 20 mg/mL and dried under nitrogen gas flow and then under vacuum for 1 h. D_2_O was added to the dried lipid films to reach a concentration of 100 mg/mL. DMPC unilamellar vesicles were prepared by extruding the lipid suspension through a heated mini-extruder (Avanti Polar Lipids) containing porous polycarbonate membranes with a pore diameter of 100 nm a total of 21 times at 60 °C and were used as “neat samples” following further dilution to 1 mg/mL. Two different types of DMPC unilamellar vesicles were prepared: (i) d_54_-DMPC in D_2_O to be used for the IL-doped cases and (ii) d_54_-DMPC:h-DMPC (9:1 molar ratio) in D_2_O to be used for the neat cases. For the “IL-doped samples”, [bmim][Cl] was then added to reach a 0.1 M IL concentration, and the samples were allowed to equilibrate for 1 h before use. The samples were stored at 60 °C until the measurements. The sample quality and stability were confirmed using dynamic light scattering, which gave the same hydrodynamic radius for neat and IL-doped samples. This is in line with a set of previous studies showing that, below their CMC, ILs do not alter the overall bilayer structure.^52^ These earlier investigations include differential scanning calorimetry studies showing that, at these concentrations, [bmim]-based ILs reduced the DMPC main gel-to-fluid phase transition temperature by 1 to 2 degrees only.^63,64^

SANS measurements were performed using the NGB 30 m SANS instrument at NIST Center for Neutron Research.^108^ The scattering vector range of 0.002 Å^–1^ < *Q* < 0.4 Å^–1^ was covered, in which , where λ and θ are the incident neutron wavelength and scattering angle, respectively. To do so, the incoming neutron wavelength was fixed to 6 Å (wavelength spread of 13.8%), and data were collected at three different sample-to-detector distances, i.e., 1, 4, and 13 m. The samples were contained in 1 mm path-length quartz cells and were measured at four temperatures in the range of 10–40 °C. The temperature was controlled with a recirculation bath with an accuracy of 0.1 °C. Data reduction was performed using the Igor Pro reduction macros supplied by NIST to correct for detector sensitivity, instrumental background, empty cell scattering, sample transmission, and solvent background, providing 1D I(*Q*) vs *Q* SANS profiles.^109^ Data analysis was performed with the SasView software package.^110^

## Results and Discussion

### Results

**Figure 1:**
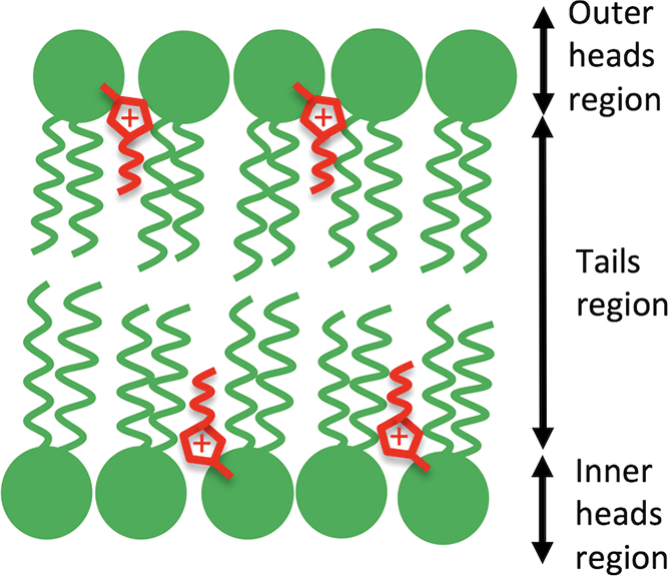
Cartoon of an IL-doped lipid bilayer, with ILs in red and lipids in green. The system has been modeled with a core multishell model, where the bilayer structure was described by three shells corresponding to (i) outer lipid headgroups, (ii) lipid tails, and (iii) inner lipid headgroups. To determine the IL absorption with reasonably good accuracy, the experiment was designed to have a high neutron scattering contrast between ILs and lipids in the lipid tail region.

Taking advantage of the neutron scattering contrast in our samples, the unilamellar lipid vesicles were modeled using a form factor for polydisperse sphere with a core and three shells containing (i) a D_2_O polydisperse core, (ii) an inner lipid head layer, (iii) a lipid tails double layer, and (iv) an outer lipid head layer exposed to the solvent, as sketched in Figure 1. The scattering intensity from dilute vesicles using a polycore three-shell model is given by^111^
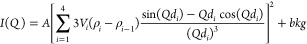
1where *A* is the scaling factor; *bkg* is the constant incoherent background; and *V*_*i*_, *d*_*i*_, and ρ_*i*_ are the volume, thickness, and scattering length density (SLD) of the core and the three shells, respectively.

**Figure 2:**
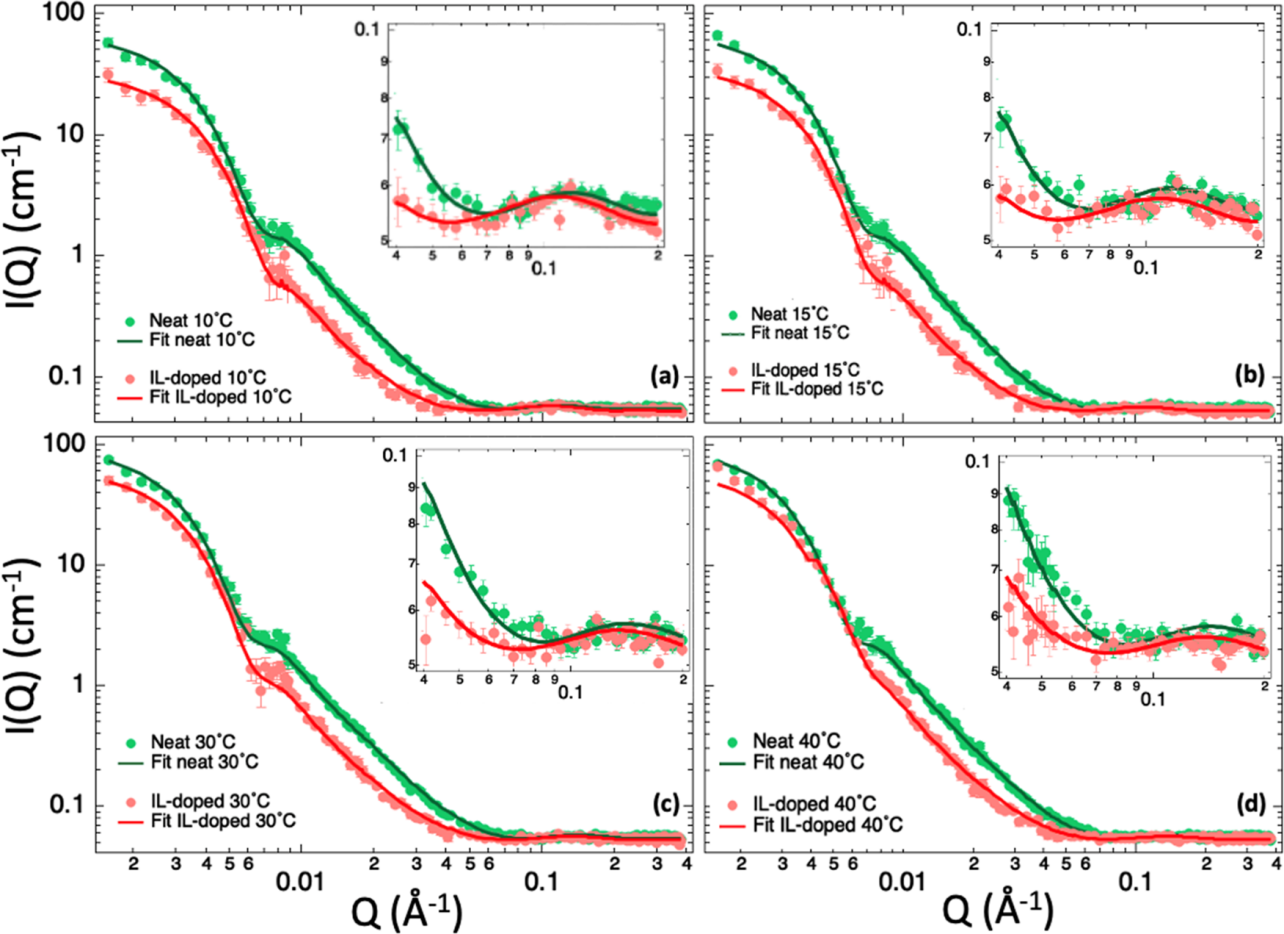
SANS data (circles) collected on neat (green) and IL-doped (red) tail-deuterated DMPC lipid vesicles at (a) 10 °C, (b) 15 °C, (c) 30 °C, and (b) 40 °C together with the fitting curves obtained using the polydisperse core three-shell model of eq 1. Fit results are reported in Table 2. The inset shows the zoomed view of the 0.04–0.2 Å^–1^*Q*-range region. Error bars represent one standard deviation.

The data were fit by eq 1, and the thicknesses of both outer and inner head regions and of the tail region of the bilayer were fixed to well-accepted values. More specifically, (i) for the fluid phase conditions (i.e., *T* = 30 and 40 °C), the thickness measured in our previous neutron reflectometry experiments on supported neat DMPC lipid bilayer^61^ was used for *T* = 30 °C and accordingly corrected to take into account the thermal expansion of the lipids at *T* = 40 °C;^112^ these values were also in good agreement with other literature values;^113^ (ii) for the gel and ripple conditions (i.e., *T* = 10 and 15 °C), other literature values^114,115^ were used to take into proper account the effect of the temperature. These values are given in Table 2. In the fits of the IL-doped cases, the thicknesses of the headgroup and tail regions of the bilayer were fixed to the neat-case values. This choice was motivated by the fact that the few angstrom variations in the bilayer thickness measured on the same system in our previous neutron reflectometry experiments could not be resolved in the SANS data;^61^ this assumption, however, may not be valid for other lipids and ILs.^116^ To take into account that the vesicle radius was not the same across the whole vesicle population, we have performed our fits by convoluting eq 1 with a Gaussian distribution of vesicle radii. This has been done in practice by incorporating a core radius polydispersity ratio, which was 0.3 for all of the fits and in line with previous SANS studies. Figure 2 shows the experimental data along with the associated fits for all of the measured conditions. By simply looking at the figure, a difference between neat and IL-doped systems was clearly visible at all of the measured temperatures, meaning that there was a measurable effect of the IL on the lipid bilayer composition.

**Table 2:** Lipid Bilayer Structural Parameters Obtained by Fitting the SANS Data of Neat and IL-Doped DMPC Unilamellar Vesicles at 10, 15, 30, and 40 °C^a^

fit parameters	neat 10 °C	IL-doped 10 °C	neat 15 °C	IL-doped 15 °C	neat 30 °C	IL-doped 30 °C	neat 40 °C	IL-doped 40 °C
inner head layer thickness, *t*_1_	10*	10*	10*	10*	9.6*	9.6*	9.6*	9.6*
inner head layer SLD, *SLD*_1_	2.8 (0.3)	3.9 (0.2)	3.5 (0.3)	4.4 (0.3)	3.0 (0.8)	4.9 (0.4)	3.0 (0.8)	4.9 (0.6)
tail layer thickness, *t*_2_	34*	34*	34*	34*	29*	29*	28*	28*
tail layer SLD, *SLD*_2_	6.51 (0.05)	6.82 (0.05)	6.42 (0.04)	6.69 (0.04)	6.29 (0.05)	6.54 (0.03)	6.29 (0.05)	6.5 (0.03)
outer head layer thickness, *t*_3_	10*	10*	10*	10*	9.6*	9.6*	9.6*	9.6*
outer head layer SLD, *SLD*_3_	3.0 (0.3)	4.3 (0.2)	3.7 (0.3)	5.0 (0.2)	3.4 (0.9)	5.1 (0.4)	3.4 (0.9)	4.9 (0.5)
*χ*^2^/*N*	1.5	1.1	1.5	1.0	1.7	1.5	1.5	1.5

a The uncertainties, reported in parentheses, are standard deviations. The “stars” represent parameters that were fixed to the literature value during the analysis. All of the lengths are in Å, and the SLD are in 10^–6^ Å^–2^. The core radius polydispersity ratio is 0.3 for all of the fits.

The fit results are reported in Table 2 for both the neat and IL-doped samples at the four investigated temperatures. As discussed above, the thicknesses of outer and inner lipid head regions and of the tail region for the IL-doped samples were fixed to the neat values. The rationale behind this choice is that previous neutron scattering experiments,^61^ as well as computer simulations^62^ have shown that low concentrations of the short-tail [bmim][Cl] IL induce a small 1–3 Å decrease of the bilayer thickness only, and this small decrease could not be resolved within the resolution of the SANS data. As a result, our analysis assumed that the bilayer thickness was not affected by the ILs. The validity of this assumption was confirmed by looking at the associated errors. The uncertainty on the overall bilayer thickness did not change between the neat and associated IL-doped samples, and its average across all samples and conditions was 5 Å, which is a common uncertainty in values extracted from modeling SANS data and of the same order of thickness variation expected to be triggered by the presence of ILs in the lipid bilayer.

**Figure 3:**
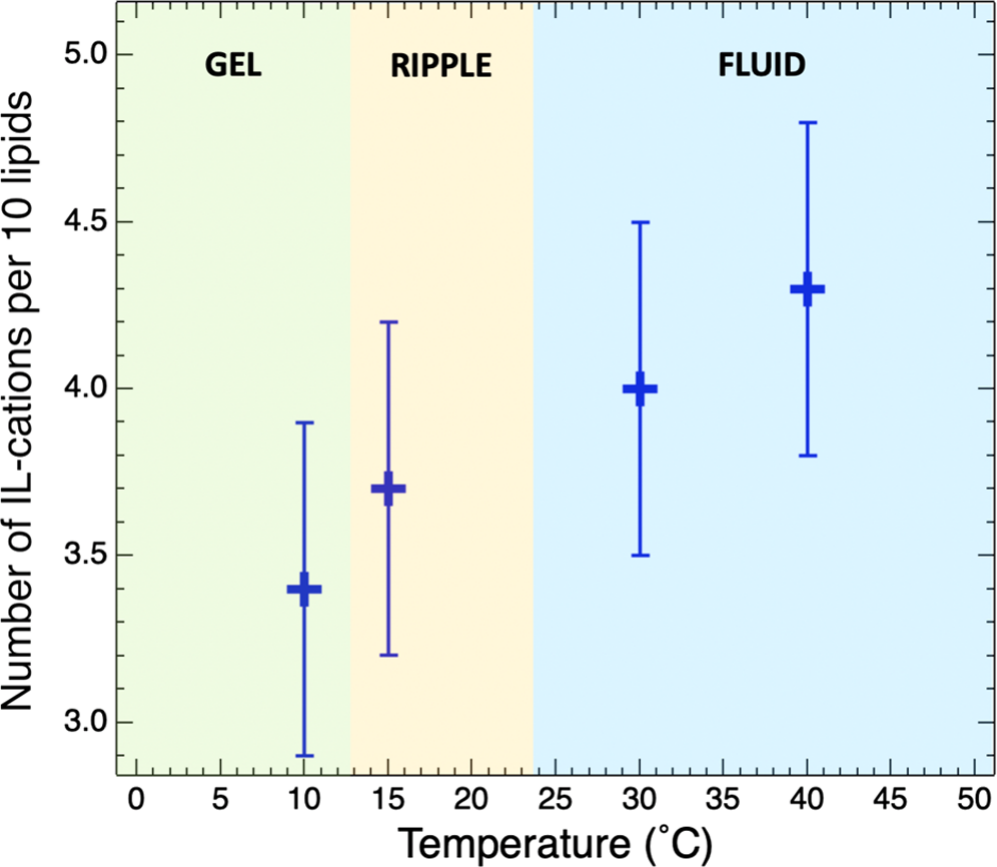
Number of IL cations per 10 lipids absorbed in the lipid bilayer as a function of the temperature, across gel, ripple, and fluid lipid phases.

Holding all thicknesses fixed at the neat values, the aim of our SANS experiments was to measure the variation in the SLDs of the lipid bilayer induced by the IL and quantify the number of absorbed ILs. In doing so, we focused on the fit values of the SLD of the tail region, as there was the greatest neutron scattering contrast between the lipid tails and ILs, i.e., 7.1 × 10^–6^ Å^-2^ vs 0.9 × 10^–6^ Å^–2^, respectively (Table 1). The head region, contrarily, is highly influenced by the hydration water and, for probing the effects of the IL in there, more detailed modeling and additional data at different SLD contrasts would be needed. By looking at the results of the fitting reported in Table 2, it is possible to conclude that the presence of the IL altered the SLD of the lipid tail region in a measurable manner at all of the investigated temperatures. Since the [Cl]^−^ anion was neither properly resolved by SANS nor was it expected to be able to penetrate the lipid tail hydrophobic region (due to its high coordination with water molecules), the observed variation in the SLD was attributed to the presence of IL cations in this region. The rationale was as follows. In the neat case, the lipid tail region only contained the lipid tails and was described by the “neat” SLD. If IL cations diffused into the lipid tail region, the scattering length density contrast of this region would change depending on the relative amounts of IL cations and lipids. As a result, (i) by computing the volume of the lipid tail region from the associated SLD measured for the neat sample and (ii) by assuming the additivity of lipid and IL volumes (i.e., the ideal mixing approximation), the number of IL cations absorbed in the lipid region was computed from the SLD of the lipid tail region measured for the IL-doped sample. The changes in the SLD accounted for approximately three and four IL cations every 10 lipids at 10 and 40 °C, respectively (Figure 3 and Table 3).

**Table 3:** Number of [bmim]^+^ IL Cations Absorbed in the DMPC Lipid Bilayer at Different Temperatures Covering Gel, Ripple, and Fluid Lipid Bilayer Phases^a^

temperature (°C)	lipid phase	IL cations/10 lipids
**10**	gel	3.4 (0.5)
**15**	ripple	3.7 (0.5)
**30**	fluid	4.0 (0.5)
**40**	fluid	4.3 (0.5)

a The uncertainties are reported in parentheses and represent one standard deviation.

### Discussion

The absorption of IL cations into lipid bilayers has been reported in a few earlier experimental and computational studies; however, these studies have been primarily focused on lipid bilayers in their fluid phase. Notably, our SANS results of the fluid phase (i.e., 30 and 40 °C) are in good agreement with these earlier studies, including our own previous investigations performed by neutron reflectivity^61^ and by full-atom molecular dynamics simulations.^62^ However, to the best of our knowledge, neither the IL absorption in lipid bilayers in their gel phase nor its temperature dependence have been investigated. The temperature range used in our SANS investigation here presented has been chosen to cover gel, ripple, and fluid phases of the DMPC lipid bilayers, with the aim to allow us to shed light on both IL absorption in gel-phase lipid bilayers and its temperature dependence. From Figure 3, it is possible to conclude that the IL cations are also absorbed in the gel and ripple bilayer phases. This is the first major result of our SANS study. This is in agreement with two recent studies showing that ILs can affect lipid bilayer properties also in their gel thermotropic phase.^116,117^

The second major result is the temperature dependence of the IL absorption within the fluid phase. Even though the error bars are large, the trend in Figure 3 suggests that the number of IL cations absorbed in the lipid region increases with increasing temperature. Since the effect of IL on lipid bilayer properties (e.g., on bilayer elasticity) is expected to be positively correlated with the number of ILs absorbed in the lipid phase, this trend suggests that the IL-induced effects will be positively correlated with the system temperature. This is in agreement with a neutron spin-echo (NSE) study we have recently carried out on DMPC lipid vesicles doped with the [bmim][Cl] IL.^66^ In this NSE study, it was shown that the IL increased the bending rigidity of the lipid vesicles and that this increment increased with temperature. In light of the SANS results presented here, we can now link the bilayer bending rigidity increase with the temperature observed earlier by NSE^66^ to an increase in the number of ILs diffused into the lipid region with the temperature. More generally, if the temperature dependence of IL absorption in Figure 3 is also seen for other lipid and IL combinations, varying the temperature can offer a good handle to control the IL absorption and, in turn, the effect of IL on biomembranes.

**Figure 4:**
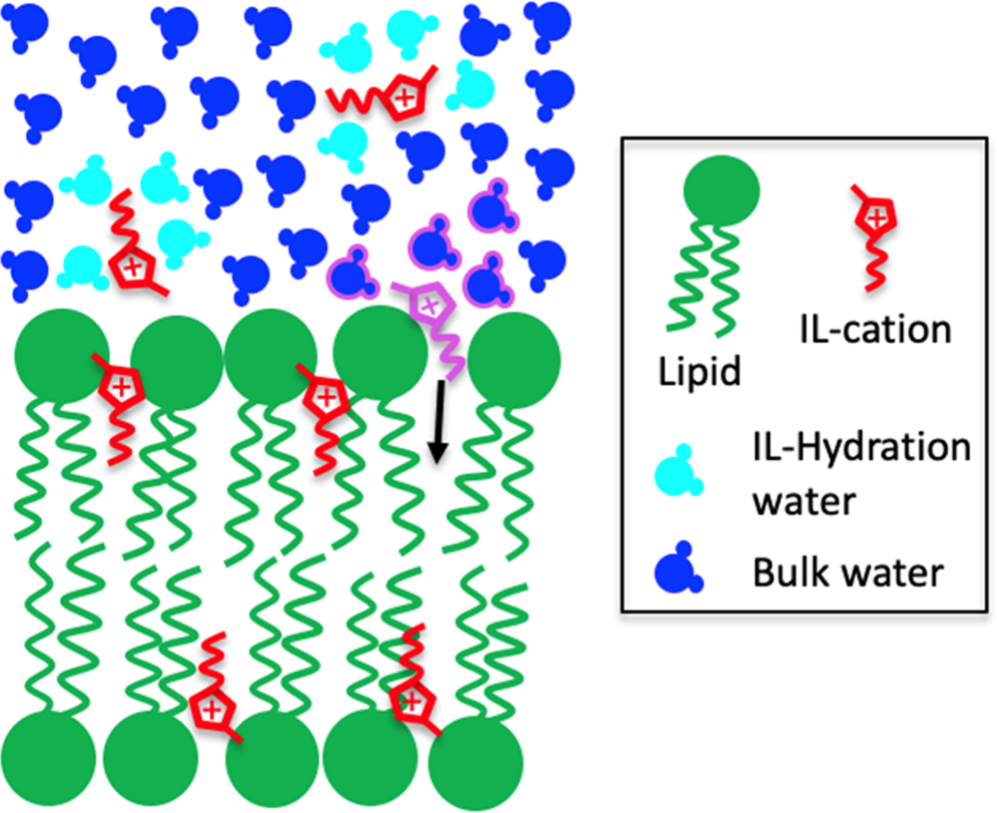
Sketch of the IL hydration water entropy-driven absorption mechanism proposed to explain the observed increase in the number of IL cations absorbed in the lipid fluid phase with increasing temperature. When the purple-highlighted IL cation diffuses into the lipid bilayer, its pink-circled hydration water molecules get released into the bulk solvent. The entropy variation associated with the release of these water molecules is positive and could be the driving force governing the behavior observed in Figure 3.

One possible reason behind this observed behavior is that there is an entropic contribution to the system free energy, favoring the configuration in which the IL cation is inserted into the lipid region rather than the configuration which is fully exposed to the solvent. Even though the configuration with the IL cation placed in the lipid region would appear the most ordered configuration, the associated overall entropy of the system could still be higher in this configuration than in the less ordered configuration in which the IL cation is in the solvent. In this case, the source of entropy could come from the water molecules forming the IL hydration shells. More specifically, while in solution, the IL is surrounded by water molecules forming its hydration layer, and the removal of these water molecules increases the overall entropy of the system (Figure 4). In summary, the temperature dependence of the IL absorption into the lipid bilayer in its fluid phase reported in Figure 3 could be driven by the IL hydration water entropy gain, as similarly observed for several other biophysical processes, including tubulin polymerization and protein fibrillation.^118−122^

## Conclusions and Remarks for the Future

To summarize, the absorption of a model IL, [bmim][Cl], in unilamellar DMPC lipid vesicles was successfully studied by means of SANS at a few selected temperatures, chosen to cover the gel, ripple, and fluid phases of the lipid vesicles. The results showed that (i) the IL cations were absorbed in the lipid bilayer also in its gel and ripple phases and (ii) the number of ILs inserted into the lipid phase increased with increasing temperature, changing from three to four IL cations for each 10 lipids at 10 and 40 °C, respectively. Tentatively, an explicative hypothesis based on the entropy gain from the IL hydration water was suggested to explain the observed temperature trend in the fluid phase, which needs further studies to be corroborated. The ability to control IL absorption with temperature can be used as a handle to tune the effects of ILs on biomembranes, for example, on their mechanoelastic properties,^45,66^ and can be exploited in bio-nanotechnological applications.^35^

SANS proves to be a powerful technique for the study of IL absorption in lipid bilayers. Future studies quantifying IL absorption should focus on different lipids and ILs. As far as ILs are concerned, [C*_n_*mim]-based ILs would offer the possibility to study the effects of IL chain length on the IL absorption. Other studies should focus on the effects of the IL anion and consider magnetic ILs^123,124^ and ILs based on amino acids,^125^ both of interest in bio-nanotechnology. On the lipid side, zwitterionic, charged, saturated, and unsaturated lipids of different chain lengths as well as ionizable lipids^126^ should be considered. In parallel to temperature dependence, future studies should consider the effect of the IL concentration in solution on IL absorption in the bilayer, from which the mixing free energy can be also computed. Moreover, the effect of IL water nanodomains should be also taken into account.^127^
